# Supplementary material for: Splice-Junction-Based Mapping of Alternative Isoforms in the Human Proteome
Source: Cell Rep. Author manuscript; Available in PMC 2020 Jan 15. (PMC6961840; doi:10.1016/j.celrep.2019.11.026)
Supplement: 2 [file NIHMS1546469-supplement-2.zip › DF1/PXD009737/TestisGluC/TestisGluC_30_DMD_LNHRFAAISHRIKTGKNEDNE.pdf]

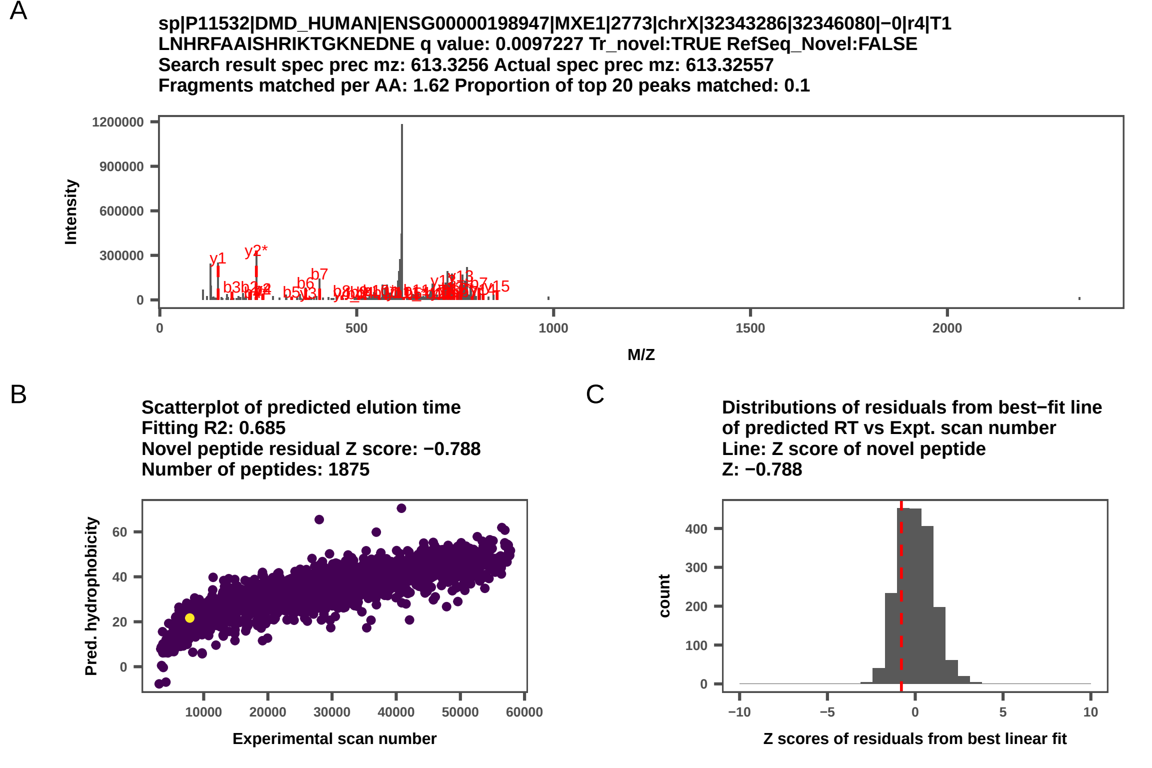
A
sp|P11532|DMD_HUMAN|ENSG00000198947|MXE1|2773|chrX|32343286|32346080|−0|r4|T1
LNHRFAAISHRIKTGKNEDNE q value: 0.0097227 Tr_novel:TRUE RefSeq_Novel:FALSE
Search result spec prec mz: 613.3256 Actual spec prec mz: 613.32557
Fragments matched per AA: 1.62 Proportion of top 20 peaks matched: 0.1
Intensity
1200000
900000
600000
300000
0
y1
y2*
b7
b6
b5
y13
y15
M/Z
0
500
1000
1500
2000
B
Scatterplot of predicted elution time
Fitting R2: 0.685
Novel peptide residual Z score: −0.788
Number of peptides: 1875
Pred. hydrophobicity
60
40
20
0
10000
20000
30000
40000
50000
60000
Experimental scan number
C
Distributions of residuals from best−fit line
of predicted RT vs Expt. scan number
Line: Z score of novel peptide
Z: −0.788
count
400
300
200
100
0
−10
−5
0
5
10
Z scores of residuals from best linear fit
